# TiO_2_ Nanoparticles Trigger Gut-to-Gill Bacterial Translocation and Dysbiosis in Zebrafish

**DOI:** 10.3390/ijms27115036

**Published:** 2026-06-02

**Authors:** Chi-Cheng Li, Der-Shan Sun, Te-Sheng Lien, Guan-Ling Lin, Ching-Feng Cheng, Kuo-Wang Tsai, Wen-Sheng Wu, Chi-Tan Hu, Ming-Der Lin, Wen-Ying Lin, Chin-Hao Yang, Je-Wen Liou, Hsin-Hou Chang

**Affiliations:** 1 Department of Hematology and Oncology, Buddhist Tzu Chi General Hospital, Buddhist Tzu Chi Medical Foundation, Hualien 970, Taiwan; kevinlcc1234@gmail.com; 2 Department of Molecular Biology and Human Genetics, Tzu Chi University, No. 701, Sec. 3, Chung-Yang Road, Hualien 970, Taiwan; dssun@gms.tcu.edu.tw (D.-S.S.); alan211@mail.tcu.edu.tw (T.-S.L.); guan0223@gmail.com (G.-L.L.); mingder@gms.tcu.edu.tw (M.-D.L.); 3 Department of Pediatrics, Taipei Tzu Chi Hospital, Buddhist Tzu Chi Medical Foundation, New Taipei City 231, Taiwan; chengcf@gms.tcu.edu.tw; 4 Institute of Biomedical Sciences, Academia Sinica, Taipei 115, Taiwan; 5 Department of Research, Taipei Tzu Chi Hospital, Buddhist Tzu Chi Medical Foundation, New Taipei City 231, Taiwan; tch33225@tzuchi.com.tw; 6 Division of General Surgery, Department of Surgery, Hualien Tzu Chi Hospital, Buddhist Tzu Chi Medical Foundation, Hualien 970, Taiwan; wuws@gms.tcu.edu.tw; 7 Department of Laboratory Medicine and Biotechnology, College of Medicine, Tzu Chi University, Hualien 970, Taiwan; 8 Research Center for Hepatology, Department of Gastroenterology, Buddhist Tzu Chi General Hospital, Buddhist Tzu Chi Medical Foundation, Hualien 970, Taiwan; chitan.hu@msa.hinet.net; 9 Center for Herbal Medicine and Nature Products Research, Tzu Chi University, Hualien 970, Taiwan; 10 Institute of Medical Science, Tzu Chi University, Hualien 970, Taiwan; 111353104@gms.tcu.edu.tw; 11 Department of Biochemistry, School of Medicine, Tzu Chi University, Hualien 970, Taiwan; 12 Smart Biomedical Chip Research Center, Tzu Chi University, Hualien 970, Taiwan

**Keywords:** nanotoxicology, titanium dioxide (TiO_2_) nanoparticle, host–microbe interactions, aquatic microbiome, microflora, metagenomic analysis

## Abstract

Titanium dioxide nanoparticles (TiO_2_-NPs) are widely produced and persist in aquatic ecosystems, yet their indirect effects on host–microbe interactions remain poorly defined. By using zebrafish (*Danio rerio*) as a sentinel species, this study investigated the effects of subchronic 5 mg/L TiO_2_-NP exposure. Dynamic light scattering was utilized to characterize the bimodal aggregates (peaks at 917 and 46,841 nm; surface charge: +22.08 mV) that define the environmental state of TiO_2_-NPs. Parallel 16S rRNA metagenomic profiling on Day 6, prior to mortality, revealed profound gut dysbiosis. A marked increase in Chao1 richness (*p* < 0.01), alongside a catastrophic 333-fold reduction in beneficial *Cetobacterium* and an 856-fold enrichment of pathogenic *Mycobacterium*, was observed. Beta-diversity and hierarchical clustering analyses revealed a striking convergence between gut and gill microbial signatures, supporting a gut-to-gill translocation model. These results suggest that TiO_2_-NPs exposure induces intestinal dysbiosis, facilitating opportunistic bacterial migration via internal (gut–blood–gill) or external (fecal–water–gill) pathways. This study identifies dysbiosis-driven secondary infection as a novel, overlooked mechanism of nanoparticle toxicity, necessitating a shift in ecological risk assessments toward host–microbe interactions.

## 1. Introduction

Titanium dioxide nanoparticles (TiO_2_-NPs), including the industrial standard Degussa P25, are among the most widely produced and used nanomaterials globally, with immense production volumes and increasing, persistent accumulation in aquatic ecosystems via industrial and domestic wastewater. We selected TiO_2_-NPs for study because of their widespread usage and priority status in the evaluation of subchronic ecological impacts. Although often regarded as chemically inert, their unique nanoscale properties, such as high surface area and photocatalytic activity, pose unpredictable risks to aquatic biodiversity. TiO_2_-NPs are commonly found in cosmetics, paints, sunscreens, and food additives [1,2]. As a result of their extensive human use, they are increasingly released into aquatic ecosystems via industrial runoff, wastewater, and other environmental pathways. In aquatic environments, TiO_2_-NPs can accumulate and potentially generate reactive oxygen species under exposure to sunlight [3,4,5], causing oxidative stress in aquatic organisms. These effects may disrupt normal physiological functions in aquatic fauna and flora, thereby threatening biodiversity and ecological balance. Contamination with TiO_2_-NPs has been shown to harm aquatic organisms mainly through direct NP-induced toxicity [6,7]. However, the extent and nature of the potential indirect effects of these nanomaterials remain largely unexplored.

Zebrafish (*Danio rerio*) are ideal model organisms for evaluating the environmental toxicity of TiO_2_-NPs for several reasons. They are small, transparent in early developmental stages, genetically tractable, and share numerous physiological pathways with higher vertebrates, including humans [8,9,10,11]. Moreover, their rapid development, external fertilization, and easy maintenance make them well suited for high-throughput toxicological screening [8]. Importantly, zebrafish, as freshwater teleosts, are highly sensitive to waterborne pollutants, such as nanoparticles (NPs), and are widely recognized as a valuable vertebrate model for disease research [9,12,13,14]. We selected zebrafish as our biological model because of their established role as sentinel species for water quality and their well-characterized mucosal immune system, which parallels that of higher vertebrates. As freshwater teleosts, they are directly exposed to suspended NPs, offering a unique opportunity for investigating the intersection of NP-induced stress and microbial community dynamics. Zebrafish models further enable the exploration of indirect toxicological pathways, such as gut-to-gill bacterial translocation, that are critical for understanding how environmental pollutants compromise organismal health. The growing use of zebrafish in preclinical and toxicological studies [6,8,10] further supports their role as an ideal sentinel species for evaluating water quality and environmental stressors. These advantages, combined with available genomic tools and established behavioral and molecular endpoints, enable the comprehensive evaluation of TiO_2_-NP-induced toxicity in zebrafish at molecular, cellular, and organismal levels.

The direct toxic effects of NPs have been well documented in zebrafish models [15,16,17,18,19,20,21,22,23,24,25,26,27,28]. However, our previous study suggested that TiO_2_-NP-induced injuries and secondary infections by opportunistic bacteria contribute to zebrafish mortality. The microbiome profiling of infected tissues, such as the gills of TiO_2_-NP-exposed zebrafish, is essential for uncovering the indirect and potentially underestimated effects of NP exposure, with our earlier metagenomic analysis of gill samples revealing a predominance of gut-associated bacterial phyla, including Proteobacteria, Bacteroidetes, and Actinobacteria [29]. Notably, the gut microbiota had not been directly analyzed in previous investigations. In this study, we conducted the microbiome profiling of gut tissues from untreated and TiO_2_-NP-exposed zebrafish and compared the results with the gill microbiota of TiO_2_-NP-treated fish. Our findings revealed that compared with the control treatment, TiO_2_-NP exposure markedly altered the gut microbial composition. We also observed the substantially overlapping profiles of microbial communities between gut and gill samples, supporting the hypothesis that NP-induced stress may promote the translocation of opportunistic bacteria from the gut to the gills.

## 2. Results

### 2.1. TiO_2_-NP Exposure Induces Distinct Gut and Gill Microbiota Shifts in Zebrafish

**Figure 1:**
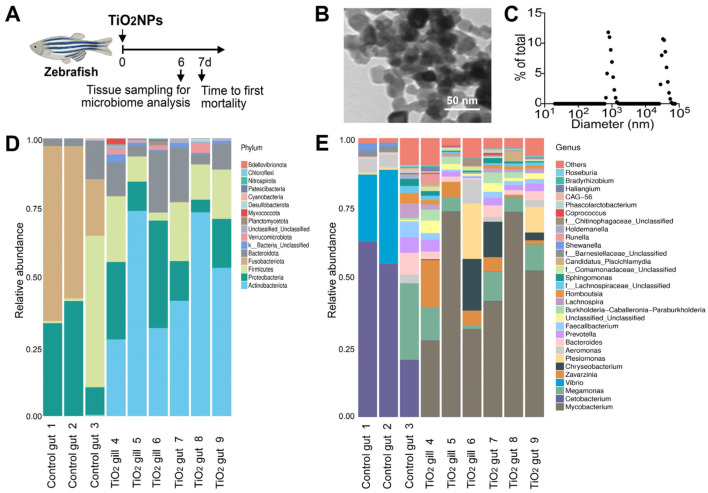
Experimental Design and Microbiota Characterization of Zebrafish Exposed to TiO_2_-NPs. (**A**) Schematic of the experimental timeline, including TiO_2_-NP exposure on Day 0, tissue sampling for microbiome analysis on Day 6, and observation of first mortality on Day 7. (**B**) Transmission electron microscopy image of TiO_2_-NPs (scale bar: 50 nm). (**C**) Hydrodynamic size distribution of TiO_2_-NPs in water measured via dynamic light scattering (DLS; ELSZ-2000) using intensity-based profiling, showing bimodal aggregation with peaks at 917 and 46,841 nm (mean size: ~25,398 nm). (**D**,**E**) Relative abundance of bacterial communities at the (**D**) phylum and (**E**) genus levels across nine samples from the untreated control gut (Samples 1–3), TiO_2_-exposed gill (Samples 4–6), and TiO_2_-exposed gut (Samples 7–9). Taxonomic profiles were determined via metagenomic analysis. Zebrafish illustration created with BioRender.com (Toronto, ON, Canada).

We performed the metagenomic profiling of gut and gill samples after six days of exposure, prior to the onset of mortality, to examine the effect of TiO_2_-NPs on zebrafish microbial communities. The experimental timeline is illustrated in Figure 1A. Representative NP morphology and size distribution were confirmed by transmission electron microscopy (H-7500, Hitachi, Tokyo, Japan) and dynamic light scattering (DLS, ELSZ-2000, Otsuka Taiwan, Taipei, Taiwan) (Figure 1B,C). Although the supplier’s datasheet described TiO_2_-NPs as ~20 nm in diameter, they readily formed large aggregates when suspended in water.

Metagenomic analysis (Figure 1D) revealed that at the phylum level, Fusobacteriota was the dominant phylum in the gut microbiota of untreated control zebrafish. Exposure to TiO_2_-NPs induced a marked shift in microbial composition, with Actinobacteriota emerging as the predominant phylum in gut and gill communities. This shift suggests a marked microbiota alteration and points to a possible shared microbial origin between the two tissues. These findings are consistent with our previous findings, which identified Proteobacteria, Bacteroidetes, and Actinobacteria as dominant phyla in gill tissues following TiO_2_-NP exposure [29].

At the genus level (Figure 1E), *Cetobacterium*, a genus associated with healthy gut function [30,31,32], was abundant in untreated control guts, whereas *Mycobacterium*, a genus associated with pathogenic responses [33,34,35], became the dominant genus in TiO_2_-NP-exposed gut and gill tissues. The shared dominance of Actinobacteriota and *Mycobacterium* across gut and gill samples points toward dysbiosis and the gut-to-gill translocation of opportunistic bacteria.

**Figure 2:**
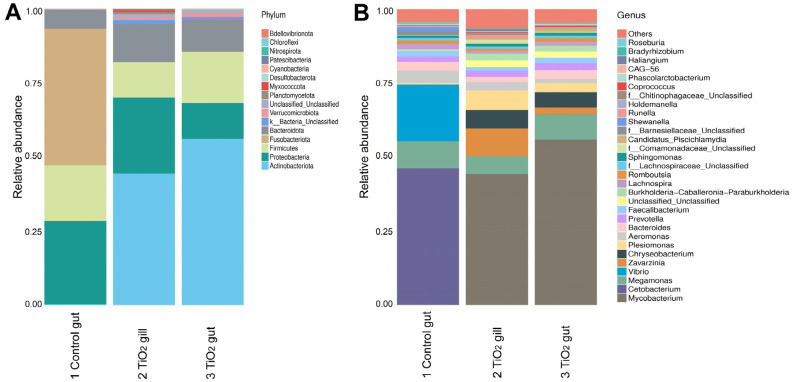
Grouped Bacterial Community Profiles at the Phylum and Genus Levels in Zebrafish Gill and Gut Samples. (**A**) Aggregated bacterial composition at the phylum level, as determined by metagenomic analysis. (**B**) Aggregated bacterial composition at the genus level, as determined by metagenomic analysis. Data represent grouped samples from untreated control zebrafish gut (Group 1: Control gut; corresponding to Groups 1–3 in Figure 1), gill samples from TiO_2_-NP-exposed zebrafish (Group 2: TiO_2_ gill; corresponding to Groups 4–6 in Figure 1), and gut samples from TiO_2_-NP-exposed zebrafish (Group 3: TiO_2_ gut; corresponding to Groups 7–9 in Figure 1).

We analyzed the grouped bacterial communities at the phylum and genus levels to assess bacterial shifts further (Figure 2A, phylum; Figure 2B, genus). Consistent with those observed in individual sample profiles, the relative abundance of Actinobacteriota (represented by *Mycobacterium*) was consistently elevated in the gut and gill microbial communities of TiO_2_-NP-treated zebrafish. This finding is indicative of TiO_2_-NP-induced microbial dysbiosis and the subsequent translocation of gut microbes to the gills. Conversely, the gut microbiome of control zebrafish maintained a dominant profile characterized by Fusobacteriota (represented by *Cetobacterium*).

**Figure 3:**
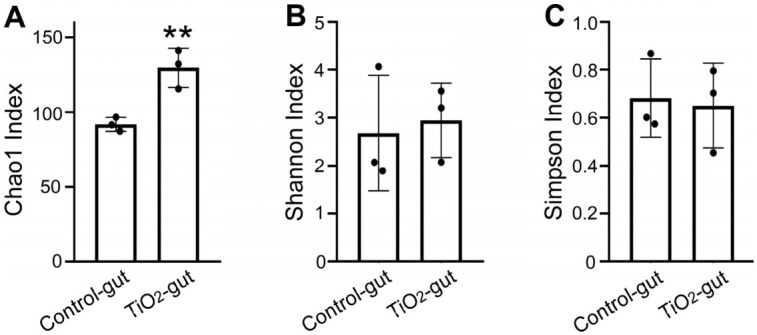
Impact of TiO_2_-NP exposure on the alpha diversity of the zebrafish gut microbiota. Alpha diversity indices were calculated to quantify changes in microbial richness and diversity following exposure to 5 mg/L TiO_2_-NPs for six days. (**A**) Chao1 index, representing estimated species richness. (**B**) Shannon index, representing microbial diversity (accounting for richness and evenness). (**C**) Simpson index, representing community evenness. *N* = 3 independent biological replicates. ** *p* < 0.01.

Alpha diversity indices, including Chao1, Shannon, and Simpson, were calculated to assess changes in microbial community complexity quantitatively. The results demonstrated that the Chao1 richness index in the TiO_2_-NP-exposed gut had significantly increased compared with that in the control gut (Figure 3A, *p* < 0.01). This increase in richness suggests that NP exposure facilitated the influx and colonization of a diverse array of opportunistic taxa in the dysbiotic gut. By contrast, the Shannon and Simpson diversity indices showed no significant differences (Figure 3B,C), indicating that while the taxonomic composition underwent a radical shift, the overall structural complexity of the community remained stable despite the overwhelming dominance of specific pathogens.

Exposure to TiO_2_-NPs induced a profound shift in the zebrafish gut microbiome, characterized by a stark divergence between potentially pathogenic and beneficial taxa. Members of the phylum Actinobacteriota (specifically the genus *Mycobacterium*) are recognized as opportunistic fish pathogens, whereas those of the phylum Fusobacteriota (specifically the genus *Cetobacterium*) are associated with health-promoting functions [30,31,32,33,34,35]. Our analysis revealed that TiO_2_-NP exposure drives these groups in opposite directions.

**Figure 4:**
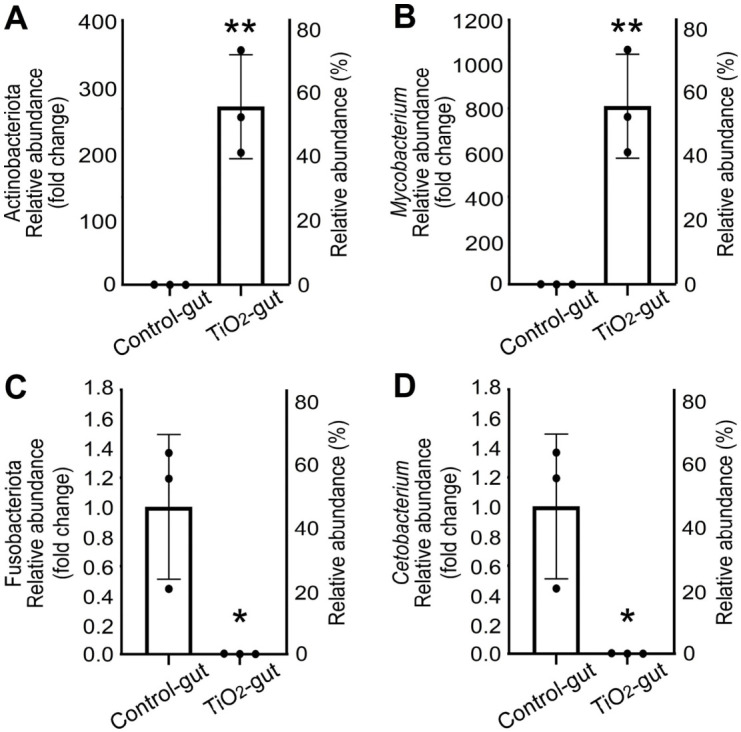
Impact of TiO_2_-NP exposure on specific gut microbiota taxa in zebrafish. Metagenomic analysis revealed significant shifts in the relative abundance of key bacterial groups in the gut of TiO_2_-NP-exposed fish (TiO_2_-gut) compared with those in untreated controls (control-gut). (**A**) The phylum Actinobacteriota markedly increased by approximately 276-fold. (**B**) At the genus level, *Mycobacterium* exhibited a marked 856-fold surge. (**C**) Conversely, the phylum Fusobacteriota showed a significant reduction, with its levels falling to approximately 0.003-fold of control levels (a ~333-fold decrease). (**D**) This decline was mirrored by the genus *Cetobacterium*, which also decreased to 0.003-fold of its original abundance. In all panels, the left y-axis represents the relative abundance expressed as fold change (normalized to the control), whereas the right y-axis indicates the percentage of the total bacterial community. *N* = 3 independent biological replicates. * *p* < 0.05, ** *p* < 0.01 vs. control gut.

Specifically, the relative abundance of the phylum Actinobacteriota surged by an astounding 276-fold (Figure 4A, *p* < 0.01). Within this phylum, the genus *Mycobacterium* exhibited a dramatic increase of 856-fold (Figure 4C, *p* < 0.01). The strikingly similar relative abundance percentages (right y-axes) between these two levels suggest that *Mycobacterium* is the dominant genus in the Actinobacteriota population following exposure.

Conversely, the phylum Fusobacteriota plummeted to a mere 0.003-fold of its original abundance, showing a 333-fold decrease (Figure 4B, *p* < 0.05). This decline was mirrored precisely by its primary constituent, *Cetobacterium*, which also fell to levels 0.003-fold of control levels (Figure 4D, *p* < 0.05). This high degree of correlation confirms that *Cetobacterium* is the major representative of Fusobacteriota in the zebrafish gut.

Given that the gut and gill microbiomes exhibited consistent bacterial patterns under TiO_2_-NP stress (Figure 1, Figure 2 and Figure 3), these findings reinforce the hypothesis of NP-induced dysbiosis and potential gut-to-gill bacterial translocation. Such substantial microbial imbalances likely serve as a key mechanism contributing to the observed physiological injury in zebrafish.

### 2.2. Clustering and Heatmap Analyses Reveal Distinct Bacterial Signatures

**Figure 5:**
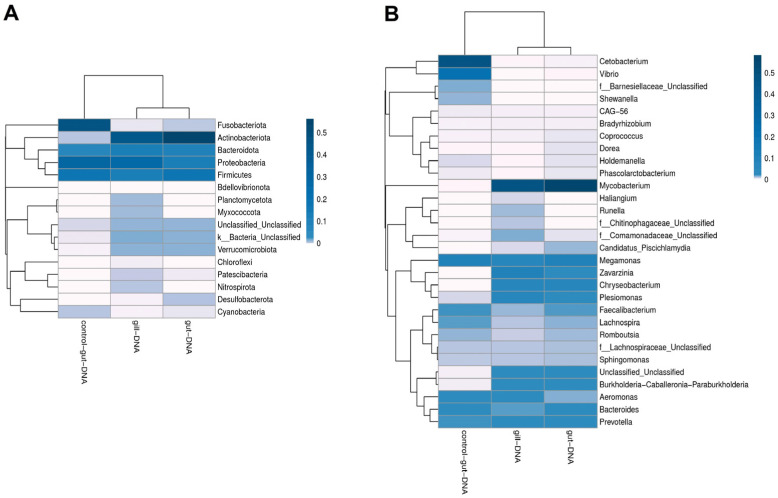
Cluster Profiles of Bacterial Abundance at the Phylum and Genus Levels in Zebrafish Gill and Gut Samples. (**A**) Clustered profiles of bacterial abundance at the phylum level in zebrafish gill and gut samples. (**B**) Clustered profiles of bacterial abundance at the genus level in zebrafish gill and gut samples. Data represent pooled samples from untreated control zebrafish gut (Group 1: control gut DNA; corresponding to Groups 1–3 in Figure 1), gill samples from TiO_2_-NP-exposed zebrafish (Group 2: gill DNA; corresponding to Groups 4–6 in Figure 1), and gut samples from TiO_2_-NP-exposed zebrafish (Group 3: gut DNA; corresponding to Groups 7–9 in Figure 1).

**Figure 6:**
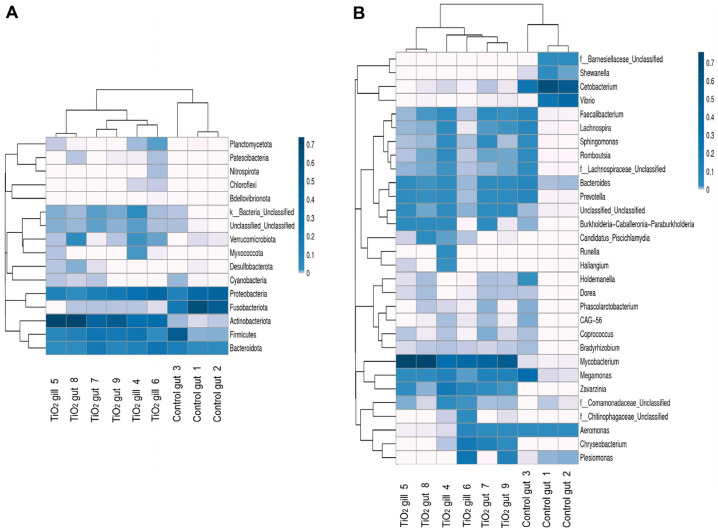
Heatmap Cluster Profiles of Bacterial Abundance at the Phylum and Genus Levels in Zebrafish Gill and Gut Samples. (**A**) Heatmap showing bacterial abundance at the phylum level in zebrafish gill and gut samples. (**B**) Heatmap showing bacterial abundance at the genus level in zebrafish gill and gut samples. Data represent grouped samples from untreated control zebrafish gut (Group 1: control gut DNA; corresponding to Groups 1–3 in Figure 1), gill samples from TiO_2_-NP-exposed zebrafish (Group 2: gill DNA; corresponding to Groups 4–6 in Figure 1), and gut samples from TiO_2_-NP-exposed zebrafish (Group 3: gut DNA; corresponding to Groups 7–9 in Figure 1).

The hierarchical clustering of bacterial abundance at the phylum and genus levels provided deep insights into microbial community structure and enabled the visualization of treatment-specific microbial signatures (Figure 5A, phylum; Figure 5B, genus) rather than focusing solely on individual taxa. TiO_2_-NP-exposed gut and gill samples clustered closely together and clearly separated from control gut samples, indicating a high degree of similarity between the treated gut and gill microbiota. These patterns were further illustrated by heatmaps (Figure 6A for phylum; Figure 6B for genus), which revealed distinct microbial profiles: one cluster of genera, including *Mycobacterium* and *Aeromonas*, was consistently enriched in TiO_2_-NP-treated samples, whereas *Cetobacterium* dominated the control group. This shift supports a treatment-induced dysbiosis that may not be evident from individual taxon analysis alone. Notably, in agreement with the results of our previous analyses (Figure 1, Figure 2 and Figure 3), Actinobacteriota and its genus *Mycobacterium* were significantly enriched in TiO_2_-NP-exposed tissues but were largely absent or present at minimal levels in controls. Together, these patterns highlight a strong correlation between gut dysbiosis and gill infection following NP exposure.

### 2.3. Beta Diversity and UPGMA Analyses Reveal Similarities Between TiO_2_ Gut and Gill Microbiota

**Figure 7:**
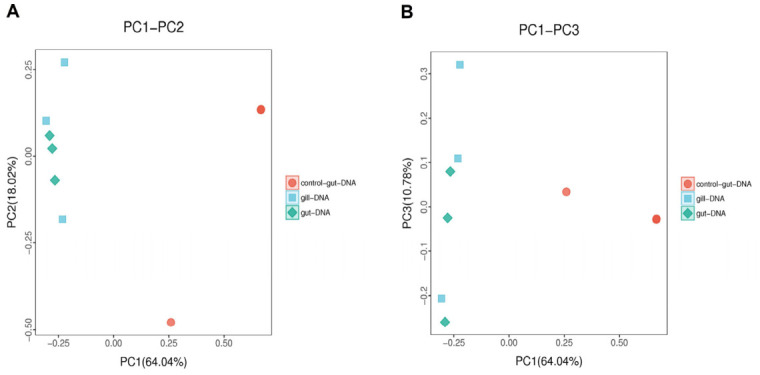
Beta Diversity PCoA Plots of Microbial Communities in Zebrafish Gill and Gut Samples. (**A**) PCoA plot showing the distribution along the PC1 and PC2 axes. (**B**) PCoA plot showing the distribution along the PC1 and PC3 axes. Data represent grouped microbiome samples from the following sources: gut samples of untreated control zebrafish (control gut DNA; corresponding to Groups 1–3 in Figure 1), gill samples from TiO_2_-NP-exposed zebrafish (gill DNA; Groups 4–6 in Figure 1), and gut samples from TiO_2_-NP-exposed zebrafish (gut DNA; Groups 7–9 in Figure 1).

Beta diversity analyses were performed using principal coordinate analysis (PCoA) (Figure 7) to quantify differences in microbial community composition. TiO_2_-NP-exposed gut and gill microbiota clustered closely along the PC1–PC2 (Figure 7A) and PC1–PC3 (Figure 7B) axes, whereas the control gut microbiota remained distinct. This convergence between TiO_2_-NP-exposed gut and gill samples suggests that gut-derived bacteria likely translocated to the gills.

**Figure 8:**
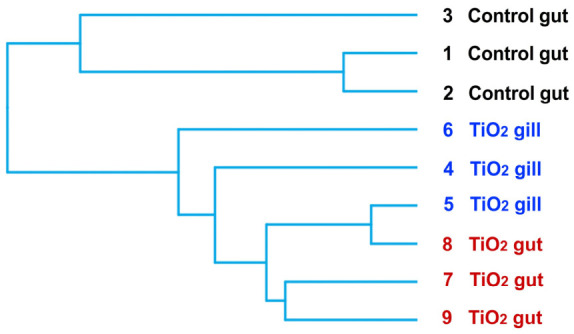
UPGMA Clustering of Microbial Communities in Zebrafish Gill and Gut Samples. Hierarchical clustering using the UPGMA method is shown. Samples are grouped as follows: untreated control zebrafish gut (Group 1: control gut [black]; corresponding to Groups 1–3 in Figure 1), gill samples from TiO_2_-NP-exposed zebrafish (Group 2: TiO_2_ gill [blue]; corresponding to Groups 4–6 in Figure 1), and gut samples from TiO_2_-NP-exposed zebrafish (Group 3: TiO_2_ gut [red]; corresponding to Groups 7–9 in Figure 1).

This finding was further confirmed by UPGMA hierarchical clustering (Figure 8), where TiO_2_ gut and gill samples grouped together, whereas control gut samples clustered separately. Together, these beta diversity and clustering results strongly support that TiO_2_-NP-induced gut dysbiosis facilitates the spread of gut bacteria to peripheral tissues, notably the gills.

### 2.4. Proposed Mechanistic Model of Gut-to-Gill Bacterial Translocation

**Figure 9:**
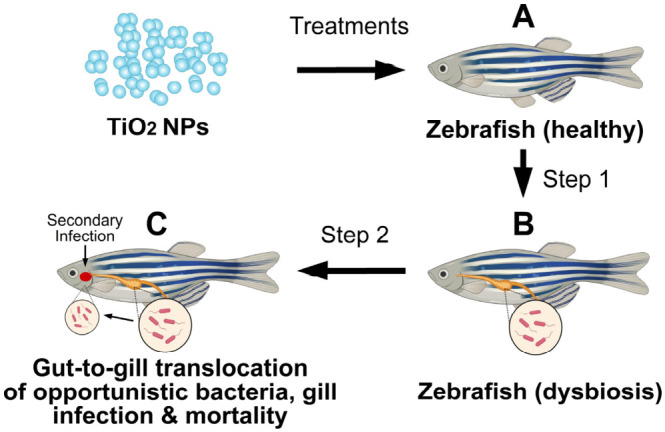
Proposed Model of TiO_2_-NP-Induced Dysbiosis and Gut-to-Gill Translocation of Opportunistic Bacteria. (**A**) Under normal physiological conditions, healthy zebrafish maintain a balanced microbial community, with the gill tissues exhibiting a low microbial burden and resistance to opportunistic microbial colonization. (**B**) Exposure to TiO_2_-NPs (treatments) disrupts this balance, inducing severe gut microbiota dysbiosis (step 1). (**C**) The resulting proliferation of opportunistic pathogens leads to their spread from the gut to the gill (step 2). This translocation may be mediated by an internal gut–blood–gill route and/or an external fecal–water–gill colonization pathway. The ensuing gill infection and tissue injury (indicated by the red icon) compromise respiratory and immune functions, ultimately leading to increased zebrafish mortality. Illustration created with BioRender.com.

On the basis of our collective findings, we propose a comprehensive model outlining the sequence of pathological events leading to zebrafish mortality (Figure 9). Under homeostatic conditions, zebrafish maintain a stable gut microbiome, with gill tissues remaining shielded from opportunistic bacterial colonization. Exposure to TiO_2_-NP triggers a profound state of gut dysbiosis, characterized by a remarkable depletion of beneficial taxa (e.g., Fusobacteriota/*Cetobacterium*) and a concurrent surge in opportunistic pathogens (e.g., Actinobacteriota/*Mycobacterium*). This microbial imbalance facilitates the spread of gut-derived pathogens to the gills potentially via two nonexclusive routes: an internal gut–blood–gill translocation pathway across damaged intestinal linings and an external fecal–water–gill colonization pathway driven by the shedding of pathogens into the aquatic environment. The subsequent colonization of gill tissues results in localized infection and inflammation, ultimately contributing to the observed mortality in zebrafish.

## 3. Discussion

### 3.1. Impact of TiO_2_-NPs on Gut Microbiota and Ecological Shifts

Zebrafish have emerged as a premier model for evaluating the multifaceted impacts of engineered nanomaterials, spanning high-precision biomedical applications [12,36,37,38,39] and large-scale environmental safety assessments [13,40,41,42]. While conventional nanotoxicological literature often emphasizes the heightened vulnerability of zebrafish embryos to TiO_2_-NPs exposure, adult cohorts frequently appear less sensitive—a discrepancy largely attributable to a historical focus on acute toxicity paradigms [43,44,45,46,47]. Indeed, standard 96 h assays yield LC 50 values exceeding 1600 mg/L [47], yet chronic exposure to significantly lower concentrations, ranging from 5 mg/L in adults [29] to ng/mL levels during early development [40], is known to trigger cumulative cellular damage and eventual mortality. We therefore employed a sub-chronic threshold of 5 mg/L to establish a relevant physiological window for observing indirect toxic effects, specifically those mediated by host–microbe interactions.

Our observations within this exposure window align with and extend findings from other metallic NP models; for instance, silver (Ag) NPs markedly alter intestinal microbial diversity in teleosts [48,49], while nickel oxide (NiO) and copper (Cu) NPs induce severe histological degradation in vital organs [50,51]. Furthermore, while conventional nanotoxicology studies often report a generalized loss of microbial diversity [52,53,54], our findings align with a growing subset of research demonstrating that NP exposure can paradoxically drive an upward trend in gut alpha diversity and species richness under specific conditions. Mirroring various NP exposures that significantly increased Chao1 and Shannon indices in marine medaka (*Oryzias melastigma*) [55], largemouth bass (*Micropterus salmoides*) [56,57], and adult zebrafish (*Danio rerio*) [58], our sub-chronic TiO_2_-NPs model induced a statistically significant increase in Chao1 richness (*p* < 0.01) in the gut, a finding that paradoxically contrasts with acute nanotoxicology paradigms.

The transition from a Fusobacteriota-dominated community in healthy guts to an Actinobacteriota-dominated profile in TiO_2_-exposed tissues marks a catastrophic disruption of host homeostasis. Specifically, our quantitative analysis revealed a 333-fold collapse of *Cetobacterium* (*p* < 0.05), a genus widely acknowledged for its salutary contributions to freshwater fish health, including nutrient synthesis and the maintenance of mucosal immunity [30,31,32,59,60]. Aligning with the Intermediate Disturbance Hypothesis (IDH) [61,62], this NP-induced stress acts as a destabilizing force on a previously stable ecosystem, disrupting the dominant commensal community. In our sub-chronic model, the depletion of this beneficial health-sentinel signifies a critical loss of colonization resistance, effectively vacating ecological niches and compromising both competitive exclusion and biological barrier integrity. This vacuum created a highly permissive environment that facilitated a subsequent massive influx and uncontrolled proliferation of diverse opportunistic environmental bacteria, most notably evidenced by an unprecedented 856-fold surge (*p* < 0.01) in the genus *Mycobacterium*. Within the context of zebrafish health, *Mycobacterium* is recognized as a primary group of opportunistic pathogens capable of inducing chronic granulomatous disease and systemic failure [33,34,35,63,64]. Unlike prior studies noting only broad microbiome shifts, our findings capture a specific pathological signature of host compartmentalization failure. Consequently, this increased richness should be interpreted not as an indicator of ecosystem health, but rather as a hallmark of severe microflora disruption and biological barrier failure, providing a distinct microbial link that supports our proposed gut-to-gill translocation axis.

### 3.2. Evidence for the Gut-to-Gill Translocation Axis

Although the 16S rRNA V3–V4 sequencing employed in this study does not possess the resolution to definitively resolve individual species such as *M. marinum* or *M. fortuitum*, the unprecedented 856-fold enrichment of the genus Mycobacterium (*p* < 0.01) provides a definitive pathological signature. In the context of zebrafish health, the genus Mycobacterium is widely recognized as a primary group of opportunistic pathogens associated with systemic mycobacteriosis [33,34,35,63,64]. Crucially, we posit that regardless of the specific species composition, a surge of this magnitude (856-fold) within a known pathogenic genus is inherently sufficient to overwhelm host mucosal defenses and trigger systemic failure. To maintain scientific rigor and avoid over-interpretation, our results are reported at the genus level—the highest resolution strictly supported by our sequencing depth. Nevertheless, the fact that this pathogenic signature was not only enriched in the gut but also became the dominant feature of the newly detectable gill microbiota reinforces the biological significance of this genus-level shift. This massive pathogenic burden, occurring in tandem with the collapse of health-sentinel taxa, serves as the primary driver of the observed mortality and provides a robust microbial link for our proposed gut-to-gill translocation axis.

It is noteworthy that the healthy control gills in our study remained ‘metagenomically invisible’ under standard sequencing protocols. This absence of a baseline metagenomic profile was a direct consequence of the negligible microbial load in homeostatic tissues, which resulted in a microbial DNA yield consistently below the technical limit of detection (LoD) required for high-quality library construction. This technical limitation serves as critical indirect evidence of the dramatic shift occurring post-exposure; while healthy gills are relatively sterile, TiO_2_-NPs stress triggered a massive microbial surge that rendered them detectable.

The fact that the newly detectable gill community so precisely mirrored the specific pathological signature of the stressed gut—specifically the shared 856-fold enrichment of *Mycobacterium*—strongly supports our proposed gut-to-gill translocation axis. Our Beta diversity (PCoA) and UPGMA clustering analyses further confirm this striking convergence, showing that TiO_2_-exposed gills and guts clustered closely together, far removed from the control group. If the observed gill infection were merely a stress-induced bloom of indigenous gill bacteria, it is statistically improbable that it would so accurately replicate the unique dysbiotic profile of the gut. Instead, this convergence suggests that the gut-derived pathogens either migrated internally via compromised epithelial barriers or were shed into the environment to colonize the gills. Thus, the transition from a metagenomically invisible healthy gill to a pathogen-dominated infected gill provides a definitive microbial link identifying secondary infection as a primary driver of NP-induced mortality.

The systemic impact of NP-induced dysbiosis observed in our zebrafish model mirrors established pathological frameworks in higher vertebrates, suggesting a conserved mechanism of organ-to-organ microbial disruption. Consistent with findings in fish, oral ingestion of TiO_2_-NPs in mammalian models exacerbates metabolic disorders by disrupting the intestinal mucus barrier, while clinical evidence from pneumoconiosis patients highlights a lung–gut axis through which inhaled particles trigger cross-organ dysbiosis and systemic inflammation [52,53,54,65,66]. By bridging gut dysbiosis with gill infection, our study identifies the gut-to-gill axis as the aquatic equivalent to these established systemic frameworks, reinforcing that NP toxicity often transcends localized tissue damage to compromise whole-organism homeostasis.

To conceptualize this systemic failure, we propose a dual-pathway model for the colonization of distal tissues (Figure 9). First, the internal gut-blood-gill route suggests that TiO_2_-NPs-induced disruption of the intestinal epithelial integrity enables opportunistic pathogens, such as *Mycobacterium*, to escape the lumen and enter systemic circulation, eventually infiltrating highly vascularized gill tissues. Second, an external fecal-water-gill pathway may function concurrently; under this hypothesis, the dysbiotic gut sheds high loads of pathogens into the surrounding water via feces—a phenomenon described as gut-mirroring the environment. As a continuous filtration interface, the gills are uniquely susceptible to secondary colonization by these waterborne, gut-derived bacteria. Whether driven by internal migration or external transmission, the resulting gill infection represents a secondary pathological consequence of gut dysbiosis rather than a random commensal adaptation.

### 3.3. Study Limitations

While our high-resolution metagenomic data definitively demonstrate a pathological convergence between gut and gill microbial signatures, several limitations remain. This study does not definitively decouple internal systemic translocation from external environmental transmission. Future research incorporating metagenomic profiling of tank water and direct assessments of bacterial loads in internal organs (e.g., blood or spleen) will be essential to validate the relative contribution of each pathway. Furthermore, identifying the specific molecular mechanisms of barrier failure, such as the role of positive surface charge (+22.08 mV) in NP-mucus interactions, will provide deeper mechanistic insights. Nonetheless, the identification of this gut-to-gill axis necessitates a shift in ecological risk assessments toward host–microbe interactions as a primary driver of NP toxicity.

## 4. Materials and Methods

### 4.1. Chemicals and TiO_2_-NPs

The chemicals utilized in this study were procured from Sigma-Aldrich (St. Louis, MO, USA). Stock solutions of Degussa P25 TiO_2_-NPs (21 ± 5 nm; Evonik Degussa, Essen, Germany) at a concentration of 1 mg/mL were prepared by dispersing the particles in distilled deionized water then performing sonication (Ultrasonic cleaner, Enshine Scientific Corporation, New Taipei City, Taiwan) at 50 W/L and 40 kHz for 20 min, as described in previous reports [3,29,67,68]. Test solutions were freshly prepared before each experiment by diluting the stock in distilled deionized water and subjecting them to the same sonication conditions for 20 min. The size distribution and surface charge (ζ-potential) of the TiO_2_-NPs in solution were analyzed through DLS with a particle analyzer (ELSZ-2000, Otsuka Taiwan, Taipei, Taiwan). The TiO_2_-NPs displayed an average ζ-potential of 22.08 ± 0.32 mV.

### 4.2. Zebrafish Maintenance and Experimental Procedures

The zebrafish (AB strain) used in this study were sourced from the zebrafish facility at the Laboratory Animal Center of Tzu Chi University, which maintains animals under specific pathogen-free conditions [69,70,71,72]. All animal procedures were reviewed and approved by the Institutional Animal Care and Use Committee of Tzu Chi University (Approval ID: 105-10). Adult wild-type zebrafish (20–30 weeks old) were maintained in a semistatic system using charcoal-filtered tap water (pH 7.0–7.4) at 28 °C ± 0.5 °C by following previously established protocols [29,36]. Lighting conditions were maintained on a 12 h light/dark cycle using incandescent bulbs (Classictone, Philips, Taipei, Taiwan) that were specifically chosen to avoid UV exposure and prevent photocatalytic effects [4,18,73,74,75,76].

Zebrafish were housed in 30 L glass tanks containing 20 L of water and fed a combination of freshly hatched brine shrimp (OSI, Snowville, UT, USA) and commercial pellet feed (Zeigler Brothers, Gardners, PA, USA). In our previous study involving TiO_2_-NP exposure, zebrafish were treated with TiO_2_-NPs (5 mg/L) in well water over a 20-day period, during which daily mortality was recorded. The first signs of mortality were observed on Day 7 [29]. Therefore, in the present investigation, microbiome analyses were conducted prior to the onset of mortality, specifically on Day 6. On Day 6 post-TiO_2_-NP (5 mg/L) exposure, zebrafish were euthanized with an overdose of tricaine methanesulfonate (MS-222, 0.03%; Sigma-Aldrich, St. Louis, MO, USA). Gill and gut tissues were then collected, weighed, and immediately stored at −80 °C for subsequent DNA extraction and microbiome profiling.

### 4.3. Microbiome Analysis

#### 4.3.1. DNA Extraction

In accordance with previously described methods [29], bacterial genomic DNA was extracted from 200 mg of frozen zebrafish gill and gut tissue samples by using the QIAamp Fast DNA Stool Mini Kit (Qiagen, Venlo, The Netherlands). The extracted DNA yield was approximately 1–2 μg per sample. DNA was stored at −20 °C prior to polymerase chain reaction (PCR) amplification. While the TiO_2_-exposed gut and gill samples yielded high-quality genomic DNA (approximately 1–2 μg per sample) suitable for downstream analysis, the microbial DNA yield from individual healthy control gills was consistently below the threshold required for successful 16S rRNA library preparation and high-throughput sequencing. This discrepancy reflects the negligible baseline microbial biomass in homeostatic zebrafish gills, compared to the massive bacterial loads induced by NP-mediated stress.

#### 4.3.2. PCR Amplification

Genomic DNA was diluted to a concentration of 20 μg/mL, and 20–30 ng of genomic DNA was used for amplification. Primers targeting the V3–V4 hypervariable region of the 16S rRNA gene were designed with Illumina overhang adapters (Illumina Inc., San Diego, CA, USA). The primer sequences were forward: 5′-TCG TCG GCA GCG TCA GAT GTG TAT AAG AGA CAG CCT ACG GGN GGC WGC AG-3′ and reverse: 5′-GTC TCG TGG GCT CGG AGA TGT GTA TAA GAG ACA GGA CTA CHV GGG TAT CTA ATC C-3′. Primers were synthesized by Mission Biotech, Taipei, Taiwan.

PCR products were purified by using GenepHlow Gel/PCR Purification Kit (Geneaid, New Taipei City, Taiwan).

#### 4.3.3. Indexing and Library Preparation

Dual indices and Illumina sequencing adapters were added to the purified PCR products by employing the Nextera XT Index Kit (Illumina Inc., San Diego, CA, USA). Final libraries were purified with AMPure XP beads (Beckman Coulter, Brea, CA, USA), resulting in a library size of approximately 630 base pairs as verified by Bioanalyzer (Agilent Technologies, Santa Clara, CA, USA).

#### 4.3.4. Sequencing and Bioinformatics Analysis

Normalized libraries were pooled and sequenced on an Illumina MiSeq platform (v3.0 chemistry), generating 250 bp paired-end reads. The resulting sequencing data have been deposited in the NCBI Sequence Read Archive under the BioProject Accession Number PRJNA1290035 (Accession Nos.: SRX29640460–SRX29640468, in which the zebrafish control gut, TiO_2_-NP-exposed gill, and TiO_2_-NP-exposed gut samples are indicated as SRX29640460–SRX29640462, SRX29640463–SRX29640465, and SRX29640466–SRX29640468, respectively). Raw sequencing reads were first processed by using Cutadapt (v1.9.1, TU Dortmund University, Dortmund, Germany) to remove adapter sequences. Quality filtering, chimera detection, and read clustering into operational taxonomic units (OTUs) were performed by using Vsearch (v1.9.6, University of Oslo, Oslo, Norway). Taxonomic classification for each OTU was assigned through the QIIME (v1.9.1, University of Colorado, Boulder, CO, USA) pipeline, utilizing the RDP Classifier (v2.2, Michigan State University, East Lansing, MI, USA) for genus-level identification and PyNAST (v1.2, University of Colorado, Boulder, CO, USA) for sequence alignment. While 16S rRNA V3–V4 metagenomic profiling provides a robust overview of microbial community shifts, its resolution for species-level identification within certain taxonomically complex genera, such as *Mycobacterium*, is technically constrained; therefore, taxonomic data are primarily reported at the genus level to ensure scientific accuracy. Alpha diversity metrics, including the Chao1 richness estimator, Shannon diversity index, and Simpson evenness index, were computed in QIIME and subsequently visualized by using R (v3.3.1, R Foundation for Statistical Computing, Vienna, Austria), SigmaPlot (v12.5, Systat Software, Point Richmond, CA, USA), and GraphPad Prism (v8.0.1, GraphPad Software, San Diego, CA, USA). These indices, derived from the OTU table, were used to assess changes in microbial community complexity. Relative abundance shifts (e.g., 856-fold increase in *Mycobacterium*) were calculated by employing Microsoft Excel (Microsoft Corp., Redmond, WA, USA) and output by GraphPad Prism (v8.0.1, GraphPad Software, San Diego, CA, USA). Statistical differences between the control and TiO_2_-NP-exposed groups were evaluated by using a two-tailed *t*-test. Beta diversity analyses, including PCoA and UPGMA clustering, were also performed by utilizing the QIIME suite to assess community similarities between the gut and gills. LEfSe (v1.0, Harvard School of Public Health, Boston, MA, USA) was employed to identify specific microbial biomarkers and differentially abundant taxa, such as *Mycobacterium*. Taxonomic hierarchy and community shifts were visualized with KronaTools (v2.7, National Biodefense Analysis and Countermeasures Center, Frederick, MD, USA) and GraPhlAn (v1.0, University of Trento, Trento, Italy).

### 4.4. Statistical Analysis

Prior to statistical analysis, data were tested for normality and equality of variances. A *t*-test was used to evaluate the statistical significance of differences in the relative abundance of bacterial taxa. *p* < 0.05 was considered statistically significant. All statistical analyses and graphical outputs were generated by using Microsoft Excel (Microsoft Corp., Redmond, WA, USA), SigmaPlot (Systat Software, Point Richmond, CA, USA), and GraphPad Prism (v8.0.1, GraphPad Software, San Diego, CA, USA). Data are presented as mean ± SD.

## Data Availability

The datasets used and analyzed during the current study are available from the corresponding author upon reasonable request.
